# Abstracts from the College of Podiatry Annual Conference 2019: Harrogate, UK. 21-23 November 2019

**DOI:** 10.1186/s13047-021-00455-x

**Published:** 2021-04-01

**www.cop.org.uk/copc19**

## SHORT PAPERS

### O01 Stakeholder views of podiatry services in the UK for people living with arthritis: A qualitative investigation

#### Catherine Bowen, Charlotte Dando, Alan Borthwick

##### School of Health Sciences, University of Southampton, Southampton, UK

###### **Correspondence:** Catherine Bowen

**Background:** Many individuals living with arthritis in the UK are not able to access NHS podiatry services to manage their foot problems. We have previously reported perceived confusion over the definition of podiatry, a podiatrist’s skill set and where podiatrists can be of most benefit within the UK NHS organisation. Uncertainty was also reported with regards to decision making around 'patient access criteria' for UK NHS health care in relation to the lower limb. The aim of this study was to explore the views of stakeholders in podiatry services, patients, commissioners and general practitioners (GP), to further understand experiences of referral, access and provision of treatment in the NHS for foot problems for patients living with arthritis.

**Methods:** To explore in-depth individual views of experiences of stakeholders in podiatry services nineteen patients, who had arthritis (osteoarthritis and/or rheumatoid arthritis) participated in one of four focus groups. In addition, seven commissioners and/or GP's took part in semi structured interviews. A purposive sampling strategy was adopted for all focus groups and semi structured interviews. To account for geographical variations, the focus groups and semi structured interviews were conducted across two predetermined regions of the UK, Yorkshire and Hampshire. Thematic analysis was employed to identify key meanings and report patterns within the data.

**Results:** Five key themes derived from the focus groups and interviews suggest a variety of factors influencing referral, access and provision of treatment for foot problems within the UK. 1. Finance (financial variations in services); 2. Podiatry scope (understanding of what podiatry services offer and traditional clinical approaches encouraging dependencies); 3. Barriers to foot care (split systems of podiatry NHS scope, notably diabetes v musculoskeletal and that ‘arthritis is invisible’); 4. Facilitators to foot care (use of NICE guidelines and stakeholder events to determine foot health service needs); 5. Systems working together (ensuring the foot health services are appropriate for the need and encouraging support for navigation of different pathways for foot health services).

**Conclusion:** The findings indicate that patients, commissioners and GPs have very similar experiences of referral, access and provision of treatment for foot problems for patients living with arthritis. Essentially, commissioners and GPs interviewed called for a transformational approach in current systems to include newer models of care that meet the foot care needs of individual patient circumstances. Patients interviewed called for better signposting and information of the different services available to help them manage their foot health needs. Through this project, we have formulated a signposting pack for all stakeholders to help them facilitate access to appropriate clinicians ‘at the right time, in the right place’ to manage foot health problems.

### O02 Patients with intermittent claudication are at a high risk of deterioration within 2 years - a longitudinal observational study

#### Anabelle Mizzi^1^, Kevin Cassar^2^, Catherine Bowen^3^, Cynthia Formosa^1^

##### ^1^University of Malta, Faculty of Health Sciences, Podiatry Department, Malta; ^2^University of Malta, Faculty of Medicine and Surgery, Mater Dei Hospital, Malta; ^3^University of Southampton, Faculty of Health Sciences, UK

###### **Correspondence:** Anabelle Mizzi

**Background:** Intermittent claudication (IC) is the most common symptom of peripheral arterial disease (PAD). It is associated with a four-fold increased risk of myocardial infarction, stroke and cardiovascular mortality. The prognosis of the affected limb is thought to be generally benign with only 10% to 20% developing symptom deterioration and 5-10% eventually deteriorating to critical limb ischaemia (CLI) within 5 years of diagnosis. However, this is thought to be an underestimation of the actual deterioration. Evidence relating to the natural history of intermittent claudication is limited. The aim of this study was to determine the outcome of patients with IC over a 2-year period.

**Methods:** A longitudinal observational study recruited patients referred for IC to a Vascular Clinic at a national teaching hospital between July 2016 and June 2017. Individuals who gave informed consent to participate were assessed for PAD by Doppler waveforms analysis, ankle-brachial pressure index, absolute toe pressures and toe-brachial pressure index. A full medical history, patient characteristics and treatments were also recorded. Patients were eligible if they were referred for the first time and were confirmed to suffer from PAD. Walking impairment was derived from a validated self-reported maximum walking distance questionnaire. Patients who had been incorrectly referred and did not have PAD as a source of their symptoms were excluded from the study.

**Results:** A total of 150 participants with IC were recruited at baseline and followed for 2 years. Seven participants were lost to follow-up and 3 died. Symptoms improved in 22% of the cohort, 14% reported stable symptoms while 64% had worsening of symptoms over 2 years with 25% deteriorating to CLI and 39% developing worse IC. Out of those who deteriorated, 54.4% (n=49) required an intervention while the rest were treated conservatively either because of non-disabling symptoms or due to unacceptably high intervention risks from other comorbidities. All participants who experienced symptom improvement had either started to exercise regularly, and/or stopped smoking. Major adverse cardiovascular and/or cerebrovascular events occurred in 13.6% of the participants. At baseline, hypertension, diabetes and hyperlipidaemia were present in 67%, 84% and 78% of the cohort, respectively. Approximately 75% were taking statins and anti-platelet therapy and 77% had a smoking history.

**Discussion:** Our findings indicate that among patients with IC, risks of symptomatic deterioration and major events is much higher (64%) than previously estimated, with a quarter of participants developing CLI within 2 years, possibly evoking the need for the recommended conservative approach to treatment of this patient group to be re-evaluated. On initial presentation patients with IC due to PAD should be closely monitored so that deterioration can be detected promptly. Effective referral pathways to specialist care should be implemented without delay in those who experience symptomatic or haemodynamic deterioration to enable early assessment of treatment options and risk-benefit assessment of intervention. Further insight into the associated risk factors at baseline in those who deteriorated in this cohort will follow in order to establish prognostic factors which would help specialist clinical decision for early intervention.

### O03 Location, type, severity and impact of foot symptoms in patients attending a specialist rheumatology clinic

#### John Tougher^1^, Lisa Newcombe^2^, James Woodburn^2^, Ruth Barn^2^

##### ^1^Dept of Podiatry Queen Elizabeth University Hospital, Glasgow, UK; ^2^Glasgow Caledonian University, Glasgow, UK

###### **Correspondence:** Ruth Barn

**Background:** Rheumatological conditions manifest in the lower limb in a variety of forms with a predilection for the joints and soft tissues resulting in pain and walking related disability. Much of the evidence is centred around rheumatoid arthritis with minimal literature on foot problems in other rheumatological diagnoses. Therefore, the overall objective of this study was to provide a comprehensive description of the foot problems occurring in a rheumatology population and a summary of podiatry interventions provided.

**Methods:** Consecutive patients attending specialist rheumatology clinics at the Queen Elizabeth University Hospital Podiatry Department, Glasgow were invited to participate. To be eligible, participants were required to have a confirmed rheumatological diagnosis and current foot problem. Demographics, disease management and podiatric intervention were recorded. The foot function index (FFI) [1] was used to record patient reported levels of foot related disability and walking speed was recorded over 10m. Self-reported disease activity was recorded using the rheumatoid arthritis disease activity index [2]. Diagnosis of foot problems and structures affected was conducted by a single experienced clinician (JT). Relationships between key variables were explored using correlation coefficients.

**Results:** A total of 53 participants were recruited, 17 male and 36 female with a mean (standard deviation) age of 55 (13) years. Of the 53 participants, 24 had rheumatoid arthritis, 5 psoriatic arthritis, 6 connective tissue disease, 5 undifferentiated inflammatory arthritis, 4 fibromyalgia, 3 adult juvenile arthritis, 3 other seronegative spondyloarthropathy, and 1 each of gout, palindromic rheumatism and haemochromatosis. The median (IQR) disease duration was 7 (2-15) years (Table 1). 23 participants (43%) had symptoms in the rearfoot, 20 (38%) had symptoms in more than one region, with 8 (15%) only the forefoot and 2 (4%) only the midfoot affected. 29 (55%) had multiple structures affected, 14 (27%) had only tendon/muscle affected, 7 (13%) only joints affected, 2 (4%) only soft tissues and 1 (2%) had only fascia affected. The majority of participants (43%) were taking at least one DMARD with 29% on combination therapy of two or more DMARDs and 10 participants (19%) were on biologic therapy. The most frequently provided intervention was a rehabilitation programme in combination with foot orthoses (57%) followed by rehabilitation programme alone (19%). Spearman’s correlation found a moderate positive relationship between total FFI and self-reported disease activity which was statistically significant r=.510 (p=0.000). A weak negative relationship was found between walking speed and self-reported disease activity r=-.271, p=0.049 indicating that as disease activity increased walking speed decreased.

**Table 1:** **(abstract O03).** Main variables.

**Variable**	**Result**
Disease duration median (IQR) years	7 (2-15)
Walking speed (m/s) mean (SD)	0.84 (0.25)
RADAI	5.4 (3.1-6.2)
FFI total	46 (28-60)
FFI pain	49 (36-65)
FFI disability	55 (34-71)
FFI activity limitation	23 (7-47)

**Conclusion:** Despite the advent of the biologics era, foot problems remain prevalent in a rheumatology population with elevated levels of foot related impairments and disability and resultant reduced walking speed. The majority of participants had multiple structures and multiple regions of the foot affected despite pharmacological intervention suggesting that both mechanical and inflammatory factors play a role in the development of foot symptoms which confirms and extends current knowledge. Further work is required to establish whether podiatry interventions are effective at reducing the burden of foot symptoms in this population.

### O04 Alterations in foot kinematics in early rheumatoid arthritis

#### Alexander Izod^1^, Catherine Bowen^2^, Kellie Gallagher^1^, Michael Seed^1^

##### ^1^School of Health Sport and Bioscience, University of East London, London, UK; ^2^University of Southampton, Faculty of Health Sciences, UK

###### **Correspondence:** Alexander Izod

**Background:** Despite advancements in the management of early rheumatoid arthritis (RA), more than half of all patients experience significant walking impairments within the first two years of diagnosis. Published research investigating the effects of early RA on foot kinematics during gait is still limited. To optimise the recognition and targeted management of early musculoskeletal pathology in RA, more comprehensive data are required. The aim of this study was to compare baseline biomechanical foot function in early RA participants to age and gender-matched healthy controls, determining explanatory relationships with measures of disease activity, physical function and disease impact.

**Methods:** In a cross-sectional study, foot kinematics were investigated using 3D motion capture in 18 participants with disease duration ≤ 2 years and 18 adults matched for age and gender. Standard clinical measures were used to collect data on disease activity, rheumatology physical function and self-reported physical impairment. Between-group differences in foot kinematics were investigated using principal component analysis (PCA). Explanatory variables of foot kinematics in early RA were investigated using linear regression analysis.

**Results:** In early RA participants, eversion of the rearfoot and midfoot was found to be significantly increased (p<0.05). Two principal components were extracted that explained 91.94% and 97.57% of the respective between-group variance in motion observed. Patterns of rearfoot eversion in early RA were explained by single regression models incorporating walking speed (R2 of 0.456, F ratio = 12.585, p = < 0.003), the footwear/impairment domain of the Leeds Foot Impact Scale (R2 = 0.358, F ratio = 8.371, p = <0.011) and rheumatoid factor (R2 = 0.358, F ratio = 8.371, p = <0.011). Patterns of midfoot eversion were explained by single regression models incorporating the timed walk test (R2 = 0.372, F ratio = 4.507, p = < 0.031), the percentage of gait at which toe-off occurred (R2 of 0.253, F ratio = 5.425, p = < 0.033) and walking speed (R2 = 0.235, F ratio = 4.912, p = < 0.042). Abduction of the rearfoot and midfoot was also found to be significantly increased (p<0.05) in early RA participants. Two principal components were extracted, explaining 97.67% and 97.2% of the respective between-group variance in motion observed. However, no variables were found to explain altered abduction angles at these sites. Dorsiflexion at the first metatarsophalangeal joint (MPJ) was significantly reduced (p<0.05) in early RA participants. A single principal component was extracted which explained 97.43% of the between-group variance observed. Reduced dorsiflexion at this site was explained by a single regression model incorporating step length (R2 = 0.372, F ratio = 4.507, p = < 0.031).

**Conclusions:** Significant alterations in the kinematics of the rearfoot, midfoot and first MPJ were observed in participants with early RA. These alterations were present despite management using current treat to target protocols. These alterations were observed to be of a greater magnitude than previously reported and were found to be largely independent of current measures of disease activity, physical function and disease impact.

### O05 Intersegmental coupling between the lower leg rearfoot and midfoot

#### Alexander Izod^1^, Catherine Bowen^2^, Kellie Gallagher^1^, Michael Seed^1^

##### ^1^School of Health Sport and Bioscience, University of East London, London, UK; ^2^University of Southampton, Faculty of Health Sciences, UK

###### **Correspondence:** Alexander Izod

**Background:** Foot kinematics in early rheumatoid arthritis (RA) have previously been investigated using discrete variable analysis of isolated joint rotations. This approach assumes that foot kinematics in early RA primarily exhibit linear relationships between data points. However, it is plausible that the coupled movement that takes place between joints exhibits non-linear motion patterns that reflect the presence of disease. These motion patterns may have gone undetected using conventional forms of data analysis and may contribute to the pathogenesis of mechanically based musculoskeletal pathology in early RA. To investigate this, the aim of this study was to analyse inter-segmental coupling of foot kinematics in participants with early RA.

**Method:** In a cross-sectional study, three-dimensional (3D) motion capture was used to investigate inter-segmental coupling relationships between the lower leg, rearfoot and midfoot in eighteen early RA participants with disease duration ≤ 2 years and 18 healthy adults matched for age and gender. Inter-segmental coupling was investigated by analysing transitions in the coordination patterns between 3D biomechanical model segments. These transitions were analysed using phase plane angles known as the continuous relative phase (CoRP). The variability component of the continuous relative phase (VCoRP) was used to assess for the presence of significant between-group differences in the coupling of motion between 3D model segments in early RA participants.

**Results:** Early RA participants exhibited a greater magnitude of frontal plane rotation at the rearfoot in relation to transverse plane rotation at the lower leg. Significant between-group differences in the VCoRP demonstrated that the variability of these motion patterns was reduced in the presence of early RA. Early RA participants also exhibited a greater magnitude of frontal plane rotation at the midfoot compared to that of the rearfoot. This was accompanied by increase in the variability of the VCoRP. A greater magnitude of transverse plane rotation at the midfoot relative to frontal plane rotation of the rearfoot was also observed. The variability of these motion patterns was found to be significantly reduced in early RA participants.

**Conclusions:** These findings suggest that significant alterations in the coupling of motion between the lower leg, rearfoot and midfoot are detectable in the first twelve months of diagnosis of RA. It is possible that altered coupling between joints may reflect additional sources of mechanically based trauma in early RA. As these data are not detectable using conventional forms of kinematic analysis, it is suggested that alterations in inter-segmental coupling should also be investigated when screening for mechanically based foot pathology in participants with early RA.

### O06 'Why feet matter': What do people really think about feet and why do we need to know?

#### Sue Skidmore

##### Centre for Health Sciences Research, University of Salford, Salford, UK

**Background:** The global health burden is now dominated by chronic non-communicable conditions such as cardiovascular disease and diabetes that can have a significant impact on foot health. This is in the context of an ageing population with widespread implications for the individual, their carers’, and the delivery of healthcare services. For a foot health treatment plan to be effective it often needs proactive patient engagement yet engaging patients in good health behaviours can be challenging. Therefore, ascertaining a deeper understanding of people’s perceptions toward feet and foot health is integral to developing impactful foot healthcare strategies to prevent a loss of foot ill health and disease progression.

Narratives can help elicit stories that may otherwise be inaccessible, increase participant engagement and empathy and can help break down defensive barriers to the uptake of health messages. Digital stories also have the capacity to promote reflective learning, improve education and enhance learning for healthcare professionals. Furthermore, the digital component ensures that access, retrieval and review of the materials is driven by the recipient and available when it is most meaningful. Some 42% of consumers have used social media to access treatment and physician reviews, 25% have posted about a health experience, and 20% have joined a health forum or community. This is in the context of a healthcare landscape able to harness the potential of the digital era to promote self-efficacy for a sustainable healthcare future.

**Method:** Digital and traditional tools were used to collect data to explore perceptions of feet and foot health across people from a broad spectrum of demographic, cultural and social backgrounds. A social media data mine was conducted to establish the content of foot-related conversations. This data was used to inform an interview schedule for one-to-one interviews and group activities with children and young people. Thematic analysis was used to analyse the data. Alongside traditional methods, digital tools were utilised to recruit participants, targeting interest groups that can typically have an interest in foot health such as sports or pathology specific open and closed groups, and the use of related hashtags in Twitter. Ethical approval was gained to disseminate video vignettes of the narratives on the ‘Why Feet Matter’ website as a health promotion resource.

**Results:** Findings at this stage indicate 4 central themes. When a person is connected to others with shared symptoms it appears to reduce the health burden; an indiscriminate trust of information sources; the clinical significance of the pathology doesn’t necessarily correlate to impact levels; and the impact on the person doesn’t always correlate with concordance with self-management.

**Conclusion:** These findings demonstrate the need to ensure verifiable healthcare sources, especially in the digital age, yet also a real opportunity to connect people with similar conditions to help alleviate the burden and encourage proactive self-management.

### O07 Let's HUDDLE

#### Donal McAteer^1^, Olivia Kingston^1^, Shauna Gamble^1^, Avril Black^2^, Jill Cundell^3^

##### ^1^Belfast Health and Social Care Trust, Belfast, Northern Ireland; ^2^Southern Health and Social Care Trust, Belfast, Northern Ireland; ^3^Ulster University / BHSCT , Belfast, Northern Ireland

###### **Correspondence:** Donal McAteer

**Background:** This service quality improvement project aimed to develop HUDDLEs within Specialist services in our Health and Social Care Trust. The Healthcare Huddle is the “*Preparatory briefing among healthcare professionals for the purpose of collaborating, exchanging information and bringing awareness to patient safety concerns*” ^1^.

HUDDLE is a mnemonic for:

H - healthcare

U- utilising

D- deliberate

D- discussion

L- linking

E- events

HUDDLEs can take many different forms and the core principles are to

- Increase situational awareness
- Change from reactive to proactive care pathways
- Improved team communication
- Allow all staff to be heard
- Create a structure for practice
- Support continuous learning

**Methods:** Small podiatry teams share caseloads and whilst advantageous in giving continuity of care, disadvantages include that sometimes staff need support and a "fresh pair of eyes" on clinical cases to provide optimum patient care. The High-Risk foot service within the hospital setting can be demanding due to the complex caseload. There was a need to improve preparation through collaboration and communication; and to establish cultural change by providing support through collective leadership thus allowing a more professional streamlined service.

**Results:** The project team and their mentor identified one of the particularly busy vascular clinics to carry out a pilot project using the HUDDLE model. A pre and post questionnaire was developed and the staff involved in this specific clinic completed this. Of the twelve podiatrists involved in this clinic, nine did not feel prepared, eight did not feel confident and five felt they were not supported. A pre HUDDLE checklist was devised. This checklist was revised after a small pilot was carried out. The HUDDLE was rolled out to all the podiatrists who work in the trust's vascular clinics. Staff have identified that HUDDLEs help improve confidence, preparation and clinical support leading to better clinical decision-making and ultimately improved the outcome for patients. This preparation makes the patient’s clinical journey more efficient and effective.

**Conclusions** Have we met our aims?

- the HUDDLE has been well received by clinicians
- it is an innovative way of working within hospital podiatry
- it has improved communication and collaboration
- create a culture of collective leadership
- improvement in patient flow, safety and efficiency

*Future development:*

- Develop an electronic version of the HUDDLE (our trust uses an electronic patient record system)
- Work with the administration team to prepare and organise HUDDLE checklists
- Incorporate time in the job plan to prepare for the HUDDLE
- Work to refine and improve the HUDDLE recognising that each one is different
- Explore how the HUDDLE can be adapted and used in the new Multi-Disciplinary Diabetic Foot clinic.

### O08 Which material is the best for my patient?

#### Grazyna Mitchener

##### Enertor, Ipswich, UK

**Background:** The number of different cushioning materials available nowadays for orthotics is mind-boggling: different grades of EVA, Poron, polyurethanes, gels, polypropylene, polyethylene, Plastazote, silicone rubbers, cork, latex, EPDM, Deleur foam, felt, etc. Each of them works in different way providing varied degree of cushioning, impact protection, energy return or combinations of all of them. They have been on the market for a while and proved to be good orthotic "working horses”. The newest scientific research provides good insight how and why these materials work. However even more exciting are recent postings from material scientists showing how to engineer new materials which don’t have some drawbacks of the traditional orthotic materials and can be purposefully tailored to orthotic applications. The material science started to investigate and mimic ingenious solutions developed already by Nature. Ever wondered how a gecko can walk safely on a ceiling, how horse hoofs protect the animal from breaking its legs even in full gallop or why soft pomelo fruit weighting 6 kg does not break up after falling from 15 m high tree? The secret of these excellent impact protection solutions lies in a specific, hierarchical structures of the natural materials, which are nano- or micro composites consisting of hard and soft domains. The aim of this investigation was to compare the behaviour of traditional orthotic materials to the new engineered ones and assess the opportunities the newcomers bring to bespoke orthotics.

**Methods**: Several different traditional orthotic materials and new bio-mimicking copolymer nano-composites were tested for the following properties: deceleration of impact force as a measure of impact protection, maximum rebound as a measure of energy returned, speed of rebound, indent under the impact force. These properties were tested for newly manufactured materials and then repeated after up to 500,000 impacts to assess the rate of materials ageing, performance deterioration due to wear and tear and ultimate compression set. The energy of the impact applied to the materials was standardised at 3 joules and the speed of the impact was 2 m/s. Materials’ hardness, density and thickness were also assessed and correlated to the impact testing results.

**Results:** The research resulted in a good mapping of different orthotic materials, including the new engineered copolymer nano-composites, for their properties important for orthotic applications. The new materials proved to possess a wide range of properties desirable in orthotics like cushioning and/or high energy return, good durability, and lightweight.

**Conclusions:** The research showed that properties of new orthotic materials can be significantly tailored by purposefully engineering their chemical composition and hierarchical physical morphology. A good knowledge of the relationships between the materials’ structure and their properties enables efficient optimisation of the materials and informed selection of materials for specific needs of any patient. This ultimately can provide patients with best performing bespoke orthotics and quick, reliable service.

### O09 The SSHeW study: Does slip resistant footwear reduce slips among healthcare workers? A randomised controlled trial

#### David Torgerson^1^, Sarah Cockayne^1^, Caroline Fairhurst^2^, Rachel Cunningham-Burley^2^, Gillian Frost^3^, Mark Liddle^3^, Michael Zand^3^, Healther Iles-Smith^4^, Catherine Hewitt^2^

##### ^1^University of York, York, UK; ^2^Centre for Health Economics, York Trials Unit, University of York, York, UK; ^3^Health and Safety Executive, London, UK; ^4^The Leeds Teaching Hospital Trust, Leeds, UK

###### **Correspondence:** David Torgerson

**Background:** Slips, trips and falls (STF) are the main cause of accidents in the workplace. They are the most common kind of reportable non-fatal injury to employees in Great Britain, accounting for nearly one million lost working days each year. Health and social care has some of the highest numbers of injuries due to STF. After consideration of workplace slip risks, employers or staff may decide to use slip-resistant footwear; however, there is limited evidence about its effectiveness. The National Institute of Health Research (NIHR) Public Health Research (PHR) Programme funded the Stopping Slips among Healthcare Workers (SSHeW) study. Its aim was to evaluate the effectiveness and cost-effectiveness of slip-resistant footwear at preventing slips among NHS employees.

**Methods:** We conducted a two-arm, pragmatic, randomised controlled trial in 7 NHS Trusts in England. NHS employees who adhered to a dress code policy and worked 22.5+ hours a week in clinical areas (e.g. wards, outpatient clinic, patients’ homes), cafeterias, food preparation/service areas or the general hospital environment were enrolled in the study. Participants were allocated in a 1:1 ratio to either the intervention group who were offered one pair of 5 star GRIP-rated slip-resistant footwear [4] or the control group who continued to wear their usual work footwear and were offered a pair of slip-resistant shoes at the end of their participation in the study. The primary outcome was incidence of self-reported slips in the workplace over 14 weeks. Secondary outcomes included time to first slip/fall, proportion who slipped/fell, incidence of falls in the workplace over 14 weeks, and cost effectiveness. Data were collected by weekly text messages and follow up questionnaires.

**Results:** Recruitment commenced in June 2017 and ended in January 2019 with 4,554 participants randomised (154 over our target sample size). Follow up was completed in April 2019. The mean age of participants was 42.7 years (range 18 to 74), 85% were female, with 80% spending most of their time on their feet and working a mean of 35.8 hours a week. Participant response to the weekly texts was high (>86%). During the pilot phase of the study, 137 out of 357 (38%) control participants reported at least one slip (90% confidence interval (CI) 34% to 43%). Data from compliance texts sent at 14 weeks report that 47% of intervention participants are wearing their shoes all of the time, 23% wearing them some of the time and 29% none of the time (the remaining 1% reported not having yet received their shoes).

**Conclusion:** Primary and secondary outcomes will be available in September 2019 and available results on clinical effectiveness will be presented at the conference.

### O10 Insoles to ease pressure (INSTEP): An offloading algorithm for the manufacture of chairside insoles for diabetic foot ulcer protection

#### Richard Collings^1^, Jennifer Freeman^1^, Jos Latour^1^, Sam Glasser^2^, Vasileios Lepesis^1^, Joanne Paton^1^

##### ^1^University of Plymouth, Plymouth, UK; ^2^Torbay and South Devon NHS Foundation Trust, Torquay, UK

###### **Correspondence:** Richard Collings

**Background:** The aim of this study is to develop an instant (chairside) offloading insole for people with diabetes featuring a novel clinical decision-making algorithm. This utilises temporal loading patterns combined with pressure patterns from in-shoe pressure measurement technology.

**Methods:** A range of methodologies informed the development of the algorithm for this instant offloading insole solution. (1) Definitive Randomised Controlled Trial data underpinned the theoretical concept and directed the utilisation of in-shoe pressure technology. (2) A systematic review identified the best type of insole and modifications for offloading. (3) A proof of concept study showed potential for implementation. (4) Expert NHS podiatrists input with context experience further informed the algorithm. (5) Patient focus groups assessed intervention acceptability.

**Results:** Together, the data collated culminated in an insole algorithm. (1) Customised chairside insoles, utilising in-shoe pressure technology, are as effective as casted Total Contact Insoles. (2) Meta-analysis identified the metatarsal dome and aperture as the best offloading modifications. (3&4) The insole protocol can be completed within 1.5 hours, utilising readily available materials and this was acceptable to patients and clinicians. (5) Patients perceived they were receiving a better insole if they saw technology being used to direct the manufacture.

**Conclusion:** *Next steps:* A mixed methods feasibility Randomised Controlled Trial is underway to evaluate the operational experience of this innovative insole algorithm to inform the design of a definitive trial.

### O11 A randomised trial of swab versus tissue sampling for infected diabetic foot ulcers: The CODIFI2 protocol

#### E. Andrea Nelson^1^, Frances Game^2^, Angela Oates^3^, Michael Backhouse^4^, Colin Everett^5^, Howard Collier^5^, Claire Davies^5^, Catherine Fernandez^5^, Benjamin Lipsky^6^, David Russell^7^, Mathew Diggle^8^, Tim Sloan^9^, Roberta Longa^10^, Sarah Brown^5^, Jane Nixon^5^

##### ^1^School of Health and Life Sciences, Glasgow Caledonian University, Glasgow, UK; ^2^University Hospitals of Derby and Burton NHS FT, Derbyshire, UK; ^3^Faculty of Health Sciences, University of Hull, UK; ^4^York Trials Unit, University of York, Uk; ^5^Clinical Trials Research Unit, Leeds Institute for Clinical Trials Research, Faculty of Medicine and Health, University of Leeds, UK; ^6^Green Templeton College, Oxford, UK; ^7^Leeds Teaching Hospitals NHS Trust, Leeds, UK; ^8^Nottingham University Hospitals NHS Trust, Nottingham, UK; ^9^Faculty of Medicine and Health Sciences, University of Nottingham, Nottingham, UK; ^10^Leeds Institute of Health Sciences, University of Leeds, Leeds, UK

###### **Correspondence:** Michael Backhouse

**Background:** Our previous research found that on average, tissue samples identified more pathogens than swabs in patients with infected diabetic foot ulcers [1]. Tissue samples are recommended in guidelines but swabs are more widely used in practice. However, it remains unclear whether using potentially more resource-intensive tissue samples alters clinician's prescribing behaviour, and ultimately if this impacts patient outcomes. CODIFI2 aims to evaluate the clinical and cost-effectiveness of tissue sampling compared to swabs on the time to healing in people with infected diabetic foot ulcers.

**Methods**: CODIFI2 is a multicentre, phase III, pragmatic, parallel-group randomised controlled trial (RCT). It includes an internal pilot, within-trial cost effectiveness (cost per QALY), sub studies evaluating culture based & molecular techniques, clinicians' perspectives on processing techniques and an approach to increasing questionnaire return. We plan to recruit 730 patients with a clinically infected diabetic foot ulcer ≥18 years from UK multidisciplinary diabetic foot clinics. Suspected osteomyelitis or an ulcer duration of ≥ 2 years are exclusion criteria.

Eligible participants will be randomised to swab or tissue sampling (1:1 ratio) via a central 24hr randomisation service. Allocation will be by minimisation with a random element using centre, index ulcer area, duration, aetiology, location and number of ulcers on both feet. The primary outcome measure is time to healing of the index ulcer. Secondary outcomes include: adherence to sampling method; healing status at 26 & 52 weeks; reduction in area at 4 weeks; duration of antibiotic prescription for the foot of the index ulcer; SAEs (including amputation, osteomyelitis, admission to hospital), and quality of life (Diabetic Foot Ulcer Scale-Short Form & EQ -5D -3L). Health economic outcomes are health resource utilisation (inc. antibiotics, cost of health and social services) and QALYs. Follow up visits will be conducted at week 4, 26, and as soon as possible after reported clinical healing for confirmation of healing by blinded assessor. Patient questionnaires and record review will be conducted at 4, 8, 12, 26, 39, 52 weeks and (for the earliest participants) 104 weeks.

**Results:** Primary analysis will be conducted on the intention to treat population. The primary end point analysis will use proportional hazards regression and the model will be fitted to time to healing with covariates for minimisation factors. Amputations and deaths will be considered as competing risks, treatment effect will be estimated with respect to time to amputation free healing. Revascularisation will be considered as a time dependent covariate. Health economic analysis will include within trial cost effectiveness (cost per QALY, using EQ-5D-3L utilities, NHS and PSS costs).

**Conclusion:** *Timescales:* Recruitment to CODIFI2 began in May 2019 and we are due to complete follow up in Oct 2022. The results for the trial will be available in 2023.

### O12 Peripheral vascular changes in the lower limbs following cocaine abuse

#### Alfred Gatt, Nicola Camilleri, Anabelle Mizzi, Cynthia Formosa

##### Faculty of Health Sciences, University of Malta, Msida, Malta

###### **Correspondence:** Alfred Gatt

**Background:** Worldwide, substance abuse is on the rise, especially amongst the young generation. Although cocaine-induced cardiovascular and cerebrovascular events are well documented, knowledge about the relationship of cocaine use and its effect on arterial perfusion in the lower limbs is scarce. Foot screening amongst this specific population is rarely conducted especially in asymptomatic patients. This study sought to investigate the relationship between cocaine use and peripheral arterial disease.

**Methods:** The study population comprised of 30 subjects’ dependent on cocaine, smoking and alcohol [Group A] and another 30 subjects’ dependent on smoking and alcohol only [Group B]. A comprehensive lower limb vascular assessment was conducted utilizing pulse palpation, Doppler spectral waveform analysis, Ankle brachial pressure index and Toe brachial pressure index to determine the arterial perfusion status in the lower limbs.

**Discussion:** The cocaine users’ group had lower ABPIs and TBPIs than the alcohol and smoking group only suggesting poorer vascular perfusion in lower limbs. Furthermore, a larger percentage of cocaine users had monophasic/continuous waveforms of all 3 pedal pulses compared to alcoholics/ smokers only. Conversely, there was a higher percentage of alcoholic and smokers only with biphasic/ triphasic waveforms compared to cocaine users implying better vascular perfusion.

**Conclusion:** The abuse and addiction to smoking, cocaine and alcohol itself are extremely problematic. Podiatric research in this field is lacking. Management and treatment of this population should not only be focused on helping individuals stop substance abuse and addictions but should also aim at identifying and treating the long-term complications which such abuses lead to. Despite the lack of symptoms such patients still share the same prognosis as symptomatic patients, showing a risk profile comparable to patients with symptomatic lower extremity PAD or with chronic heart disease. Screening and examination of lower limb perfusion will undoubtedly go a long way towards alleviating to some extent, the burden and costs related to atherosclerotic disease and its complications in this specific population.

### O13 Podiatry is an effective setting for providing opportunistic testing for atrial fibrillation (AF) using mobile ECG devices

#### Monica Fisk^1^, Nicholas Tuck^2^, Alex Lang^3^

##### ^1^Guy's and St Thomas', London, UK; ^2^Guy's and St Thomas' Community Podiatry, London, UK; ^3^Health Innovation Network, London, UK

###### **Correspondence:** Monica Fisk

**Background:** 1 in 5 strokes in the UK are caused by AF and are associated with greater disability and mortality than non-AF strokes. Early detection of AF can reduce stroke due to timely initiation and optimization of treatment, yet it is estimated that 500,000 people in the UK have undiagnosed AF. The number of people needed to test for unknown AF is approximately 1 in 71 people aged >65 (1.45%). With the advent of mobile ECG devices, it is increasingly possible to provide opportunistic testing for AF in novel settings. To determine whether podiatry is a setting where opportunistic testing for AF is worthwhile.

**Methods**: Through a National Academic Health Science Network (AHSN) project to increase detection of AF, the community podiatry team at Guy’s & St Thomas’ NHSFT submitted an expression of interest to Health Innovation Network to trial mobile ECG devices for opportunistic testing for AF within their clinical practice. Seven Kardia (Alivecor) mobile ECG devices were allocated for use in community clinics, domiciliary visits and at AF awareness events. Device usage was reported monthly by Alivecor through the national AHSN Network project.

**Results**: Between April 2018 and November 2018, 555 pulse rhythm checks using Kardia devices were performed by the podiatry team, detecting 25 people with possible AF, who were referred to their GP for further investigation. Possible AF detection was 4.5%, or 1 in every 22 people tested.

**Conclusion**: Podiatrists are well placed to detect undiagnosed AF using mobile ECG devices. The possible detection rate observed may be due to patients being older and often with existing cardiovascular risk factors. Clear communication of positive findings to those tested is key to reduce anxiety. Referral to GPs to ensure timely investigation, diagnosis and treatment is paramount.

### O14 Multimorbidity predicts poor foot health outcomes in people with musculoskeletal foot pain

#### Gordon Hendry^1^, Linda Fenocchi^2^, Helen Mason^2^, Martijn Steultjens^2^

##### ^1^Glasgow Caledonian University, Glasgow, UK; ^2^School of Health and Life Sciences, Glasgow Caledonian University, Glasgow, UK

###### **Correspondence:** Gordon Hendry

**Background**: Multimorbidity is prevalent and adversely affects health outcomes. Foot pain is common and one of the primary reasons for utilisation of podiatry services. At present, little is known about the impact of multimorbidity on foot health and related outcomes following podiatric intervention. The aims of this study were to evaluate whether there is a difference in foot health outcomes following exposure to podiatric foot care for people with and without multimorbidity; and ii) to evaluate whether the presence or absence of multimorbidity affects patients’ perceptions of change in foot pain.

**Methods:** The PROMFoot study is a prospective cohort study of adults with a new episode of foot pain attending the podiatry biomechanics service within the NHS Greater Glasgow and Clyde health board. Baseline medical comorbidity status (no condition, single condition, multiple conditions), longitudinal data on foot health measured using the Foot Health Status Questionnaire (FHSQ), and patient rating of change scores for foot pain were obtained from the PROMFoot study at baseline, and 3 and 6 months after podiatric intervention. Foot health scores (pain, function, footwear and general foot health) and perceptions of change for foot pain were compared between comorbidity groups using Kruskal-Wallis and Mann-Whitney post hoc tests. Associations between multimorbidity and foot health outcomes were evaluated using Pearson's chi-square and multivariate linear regression analyses.

**Results:** A total of 115 participants (59% female) with a mean age of 55 years were included. Multimorbidity was common, affecting 61 participants (53%); while 28 (24.3%) and 26 (22.6%) reported single or no medical comorbidities respectively. Significantly worse foot health scores for all FHSQ domains were observed for the multimorbidity group at baseline, 3 and 6 months. Change scores for foot pain were similar between groups and demonstrate modest improvements, however multimorbidity group membership was strongly associated with a perceptions of change in foot pain. Multimorbidity was independently associated with poorer foot function outcomes at 3 months, and poorer foot pain, foot function and footwear outcomes at 6 months.

**Conclusions:** Multi-morbidity is associated with poor foot health outcomes and lower rates of self-perceived improvement in foot pain following podiatric intervention for musculoskeletal foot pain.

### O15 Are knee gait kinematics and temporal data dependent upon body mass index?

#### Diana Hodgins

##### European Technology for Business Holdings Ltd, UK

**Background:** There is little published information on how knee gait kinematics vary with BMI. This is because the method of obtaining data uses an optical gait lab, which is expensive and time consuming. Studies that are published focus on joint forces, rather than joint kinematics and have small sample sizes due to the use of the optical lab1. The development of inertial sensors opens the possibility of monitoring large numbers of people in normal surroundings.

**Methods:** This paper provides gait kinematic data in the sagittal plane for 254 healthy people, age matched across the BMI range 15-35. Each person was measured using GaitSmart, an inertial sensor based system that has been validated against the optical gold standard6 and used in many gait studies, including people with knee OA and joint replacement. The inclusion criteria was no lower limb joint replacement, no diagnosis of hip or knee OA and no neurological condition that would affect their mobility. GaitSmart comprises six inertial measurement units, which contain three orthogonal gyroscopes and three orthogonal accelerometers, with accompanying Velcro straps. The sensors are. Velcro straps are applied on the lateral sides of the hip, just above the iliac crest, the thigh, just below the greater trochanter and the belly of the gastrocnemius muscle of the calf. Patients stand still for ten seconds to calibrate the sensors.

- Then each patient walks up and down a 20m corridor at their self-selected speed.
- Sensors are removed from the straps, switched off and attached back to the laptop. Dedicated software is used to analyse the data.

**Results:** Kinematic data for knee range, stance flexion and knee range symmetry, plus temporal stride duration are quoted values. Speed is calculated from other quoted gait parameters and height. The results show that there are no statistically significant differences in any of the gait parameters or speed with BMI (table 1).

**Conclusion:** It is often assumed that people with a higher BMI adapt their gait when compared to those with a healthy BMI. However, this data on 254 healthy people shows the knee joint moves the same in the sagittal plane, stride duration remains constant and speed doesn’t vary. This isn’t in contradiction to publications referring to joint loading, rather it provides additional, complimentary information. A higher BMI may predispose people to medical conditions such as osteoarthritis of the hip or knee, or may cause the disease to progress more rapidly as the loading on the joints is greater5. However, if the person remains healthy, their knee gait kinematics in the sagittal plane do not alter with BMI.

**Table 1:** **(abstract O15).** Data range collected for study.

**BMI**	**Number**	**Age**	**Speed [m/s]**	**Knee Range [Deg]**	**Average Duration [s]**	**Stance Flexion [deg]**	**Knee Symmetry [%]**
15-20	40	39.88	1.18±0.15	66.8±5.5	1.04±0.08	19.2±4.8	1.4±6.1
20-25	80	39.65	1.17±0.15	66.3±4.8	1.04±0.08	20.4±4.2	2.5±6.4
25-30	80	40.03	1.14±0.15	66.3±5.2	1.06±0.07	19.7±4.9	2.3±6.1
30-35	54	44.78*	1.16±0.19	66.1±6.2	1.05±0.07	20±6	1.4±7
* P <0.01	P-Value in comparison with BMI 20-25						

## POSTERS

### P01 Development and validation of a new tool to assess inflammatory foot disease activity in rheumatoid arthritis: the Rheumatoid Arthritis Foot Disease Activity Index

#### Anika Hoque^1^; Kellie Gallagher^2^; Anne McEntegart^3^; Duncan Porter^3^; Martijn Steultjens^4^; Jim Woodburn^4^; Gordon Hendry^4^

##### ^1^Glasgow Caledonian University / NHS Greater Glasgow & Clyde, Glasgow, UK; ^2^University of East London, London, UK; ^3^NHS Greater Glasgow and Clyde; ^4^Glasgow Caledonian University, Glasgow, UK

**Background:** The foot is commonly affected in rheumatoid arthritis (RA) resulting in pain, walking difficulties and disability. Omission of foot joints from the disease activity score-28 (DAS-28) may lead to underestimation of foot disease and suboptimal medical and non-medical management. The overall objective of this study is to evaluate the measurement properties of the Rheumatoid Arthritis Foot Disease Activity Index-5 (RADAI-F5), a newly developed 5-item (0-10 numerical rating scale) patient-reported outcome measure (PROM) with summary score (0-10) for measuring foot disease activity in people with RA.

**Methods:** Participants with RA recruited from NHS rheumatology outpatient clinics completed the RADAI-F5, a self-modified Rheumatoid Arthritis Disease Activity Index (mRADAI-5) which is a self-reported measure of global disease activity, the Foot function index (FFI), Foot Impact Scale impairment/footwear (FIS-IF) and activity/participation (FIS-AP) subscales. DAS-28 was also recorded for each participant where possible. Participants completed RADAI-F5 again at 1 week to allow evaluation of 1-week reproducibility. Construct validity was evaluated using Spearman’s rho to test a priori hypotheses for expected strength of associations between the RADAI-F5 and the alternative disease activity and foot—related disability scales at baseline. Internal consistency was evaluated using Cronbach’s alpha. One-week reproducibility was evaluated using the intra-class correlation coefficient (ICC), 90% smallest detectable change (SDC90), and 95% limits of agreement (LOA). Content validity was evaluated using 5-point Likert scales for readability and relevance.

**Results:** Of 142 respondents, 103 were female and 36 were males with a mean (SD) age of 55 years (12.5) and mean (SD) RA disease duration of 39 months (70.4). Mean (SD, range) RADAI-F5 scores for the sample were 5.02 (2.47, 9.2). Associations were largely consistent with a priori hypotheses for construct validity. Strong associations were observed between the RADAI-F5 and MRADAI-5 (0.789, CI 0.73-0.85), the FFI (0.713, CI 0.62-0.79) and FIS-IS (0.695, CI 0.66-0.82) (p<0.001). Moderately associaitons were observed with the FFI-AP (0.478, p<0.001, CI 0.37-0.63). A weak associations was observed between the RADAI-F5 and the DAS-28 (0.379, p<0.001, CI 0.26-0.57). The RADAI-F5 demonstrated high internal consistency (Cronbach’s Alpha=0.82), and floor and ceiling effects were both absent. The RADAI-F5 demonstrated good reproducibility (ICC=0.868, p<0.001, CI 0.80-0.91) and the value for SDC90 was 2.26. The upper and lower bounds for 95% LOA were -2.57 to 2.80, with 97% of scores observed within these bounds. Content validity was confirmed with 82% and 84% of participants rating the instrument as relevant and easy to understand respectively. The median time for completion was 5 minutes.

**Conclusion:** The RADAI-F5 is a highly valid and reliable PROM for measuring foot disease activity in RA patients. Furthermore, the RADAI-F5 appears to be feasible for use in clinical practice and can be used as an adjunct to the DAS28 to measure foot disease activity.

### P02 Persistent rheumatoid arthritis disease activity in the feet of people treated with biologic therapy

#### Lindsey Cherry^1^; Heidi J Siddle^2^; Ernest Wong^3^; Nigel K Arden^4^; Christopher J Edwards^5^; Catherine J Bowen^6^

##### ^1^University of Southampton & Solent NHS Trust, Southampton, UK; ^2^Leeds Institute of Rheumatic and Musculoskeletal Medicine, University of Leeds, Leeds, UK; ^3^Portsmouth Hospital NHS Trust, Portsmouth; ^4^Nuffield Department of Orthopaedic, Rheumatology and Musculoskeletal Sciences, University of Oxford, Oxford, UK; ^5^University Hospital Southampton NHS Foundation Trust, Southampton; ^6^University of Southampton, Southampton, UK

**Background:** Rheumatoid arthritis is a chronic inflammatory arthropathy that affects multiple synovial joints. In recent years the provision of biologic therapy has increased; a drug treatment that has been shown to have significant benefit over previous treatment approaches. However, choosing the best drug or achieving the optimal dose can be problematic. There is anecdotal evidence to suggest that the beneficial effect noted systemically may not be evident in the foot. As such, the aim of this preliminary study was to explore whether signs of disease activity within the forefoot of patients starting biologic therapy could be observed.

***Methods****:* A prospective, longitudinal, observational study design was undertaken, for which ethical permission was granted (ref: REC13/LO/1344). Patients with a rheumatologist confirmed diagnosis according to ACR/EULAR criteria4, of rheumatoid arthritis, for whom starting biologic therapy was indicated according to NICE criteria5, attending any of three NHS rheumatology centres, were recruited to participate. A pragmatic sample size was achieved based upon the maximum number of eligible patients recruited over one year. Participants were assessed on three occasions; prior to starting, and at 12 and 24 weeks after starting therapy.

Disease activity was calculated using the 28-joint disease activity score (DAS 28)6. A positive therapeutic response was noted if a reduction in DAS-28 of 1.2 points or more was observed. Disease remission was noted if a DAS-28 score of below 2.6 was observed. Musculoskeletal ultrasound of the forefoot was completed to detail metatarsophalangeal (MTP) joint synovitis, tenosynovitis, bursitis or the presence of forefoot bursae without Doppler signal. Foot pain was recorded as a binary outcome of either ‘present’ or ‘absent’. Patient reported foot impairment and foot related activity limitation was determined using the Foot Impact Score7.

Results: Thirty-three patients took part in this study. A summary of results is shown in table 1. At 12 and 24 weeks, 52% and 36% of patients had a DAS-28 treatment response respectively. Foot pain was still present in 64% and 70% of patients at 12 and 24 weeks respectively (including in 70.6% and 66.7% of those achieving a DAS-28 response). MTP joint inflammation was evident in 51.5% and 45.5% of patients at 12 and 24 weeks respectively (including in 41.2% and 41.7% of those achieving DAS-28 response). Foot impairment was consistent across all time points, whilst activity limitation initially improved and then worsened.

**Conclusion***:* DAS-28, foot pain and foot-related activity limitation had a similar trend of initial improvement followed by worsening. All other outcomes showed continuous improvement trends; although indicators of disease activity within the forefoot, foot pain, foot impairment and activity limitation all remained comparatively high. Despite this, the feet are not currently part of the clinical assessment required to evaluate treatment response. *Conclusion:* Signs of ongoing disease activity within the forefoot of patients starting biologic therapy were observed across all time points. The findings suggest that systemic indicators of disease activity and those observed within the foot may not directly correlate.

**Table 1:** **(abstract P02).** Summary of results for each of the outcome measures.

**Outcome measure (possible score range)**	**Baseline Count, mean ± SD (min-max)**	**12 weeks Count, mean ± SD (min-max)**	**24 weeks Count, mean ± SD (min-max)**
MTP joint synovitis (0-10)	23, 2 ± 3 (0-10)	17, 2 ± 3 (0-10)	15, 2 ± 3 (0-10)
Forefoot bursae (0-18)	29, 5 ± 4 (0-18)	20, 3 ± 3 (0-8)	20, 3 ± 4 (0-15)
Forefoot bursitis (0-18)	25, 4 ± 4 (0-18)	16, 2 ± 3 (0-8)	16, 3 ± 4 (0-17)
Tenosynovitis (0-20)	11, 1 ± 2 (0-8)	7, 1 ± 1 (0-4)	4, 0 ± 1 (0-7)
Foot impairment (0-21)	15 ± 5 (1-20)	10 ± 5 (2-20)	10 ± 5 (1-20)
Foot related activity limitation (0-30)	18 ± 9 (0-30)	14 ± 10 (0-29)	15 ± 5 10 (0-30)
Pain (Yes/No)	27 (82%)	21 (64%)	23 (70%)
DAS 28 (2-10)	6 ± 1 (4-7)	4 ± 1 (1-8)	4 ± 2 (1-8)

### P03 TRAINING the next generation of clinical rheumatology researchers: evaluation of a graduate Allied Health Professional and Nurse internship programme

#### Catherine Bowen; David Wright; Mary Fry; Joanna Adams

##### School of Health Sciences, University of Southampton, Southampton, UK

**Background;** Building research capacity is an essential part of sustaining evidence based practice in nursing and allied health professions working within rheumatology. Whilst medical and dental professions have a strong tradition of research capacity building, the situation for Allied Health Professionals and Nursing in the UK is less developed. The aim of this study was to evaluate a collaborative internship programme across five UK universities from 2015-18 (Universities of Southampton, Leeds, Salford, Oxford, the West of England). The internship included an eight-week programme of structured training workshops and research project delivery, and ongoing mentoring by experienced researchers. Sixteen interns were recruited from across the UK, including physiotherapists (7), podiatrists (5), occupational therapists (2) and nurses (2).

**Methods:** The evaluation employed mixed methods including: analysis of research metrics, an annual evaluation questionnaire sent to all 16 interns, and qualitative email interviews (12 interns, 13 mentors) conducted at the end of the internship programme. Interpretive phenomenological analysis of transcripts was used to identify recurring themes.

**Results:** Early quantitative outcomes from internship projects include three peer-reviewed publications, and 21 conference abstract presentations. Interns reported positive changes in their perceptions of research and rheumatology, including a realisation that clinical academic pathways were possible. Skills attained of most value to interns were technical research (e.g. qualitative research), research process (e.g. securing funding), rheumatology knowledge (e.g. fatigue), and general skills (e.g. communication). Two domains of impact were identified. First, the programme directly impacted on research careers with four interns securing clinical academic positions and most others reporting commitment to pursuing active research in the near future. Second, the internship had an impact on practice for those entering full-time clinical careers. Interns spoke of their ability to be ‘critically aware’, seeking an evidence base for clinical decisions. Many spoke of a new confidence in expressing opinions with clinical colleagues. Others spoke of the need for patient-centred care, learned from the Patient and Public Involvement training provided by the internship. Similarly, interns reported an increased awareness of the wider relevance of rheumatology, which influenced their subsequent clinical practice. One challenge for the internship programme identified by mentors was the difficulty in attracting interns from all Allied Health Professionals and Nursing backgrounds (notably nurses). In addition, several interns entering full-time clinical roles reported difficulties in continuing research in environments that devalued such activity.

**Conclusion:** The collaborative internship programme has been successful in supporting research capacity building by introducing newly qualified allied health professionals and nurses to research and rheumatology. This has generated tangible benefits through research outputs, clinical academic careers and influencing clinical practice. The programme has been successfully renewed for a further three years from 2018-2021 and is being informed by the evaluation findings. It has now been extended to include all UK AHP professionals (Health and Care Professions Council registerable) and nurses. Additionally, two new HEIs (Glasgow Caledonian and Keele Universities) have joined the national consortium. The programme serves as a model for research capacity building for other health conditions and professions.

### P04 The effectiveness of foot and ankle orthopaedic triage on waiting times by podiatrists

#### Andrew Cumming

##### The Royal Orthopaedic Hospital NHS Foundation Trust, Birmingham, UK

**Background**: There has been an increase in the demand for foot and ankle assessment in the specialist orthopaedic hospital. Waiting times have been increasing over the past few years and there is need to reduce this. Podiatrists to work in an extended scope practice role at the specialist orthopaedic hospital have been used to create new patient assessment slots to help with this increased demand. The podiatrists undertake the first level of paper triage and help to triage (Face- to-Face) and assess the new patient’s that come through the foot and ankle team. The main objective of the introduction of this clinic was to reduce the waiting times for new foot and ankle patients that were referred into the hospital. This would be reviewed constantly and would help inform as to if the clinic should be made permanent after the pilot phase (6 months).

**Methods:** The use of Podiatrists in foot and ankle clinics is not new (Walsh et al, 2014) however the use of Podiatrists employed by ‘tertiary’ specialist orthopaedic hospital is not well documented. The podiatrists would see the patients in new standalone clinics as they work within the consultant lead foot and ankle clinics and undertake new patient assessments, undertake treatment plans reviews, see post-operative patients and discharge patients by offering autonomous care. The innovate element of this service review is to promote the effectiveness of podiatrists who work in an extended role at reducing waiting times.

**Results:** 495 patients have seen in the 21 months from April 2017 to December 2018. 224 patients (45%) seen in the triage foot and ankle clinics have been discharged at the first appointment. 27 (6%) were referred onto surgical intervention, of which 92% went on to have surgery, and 132 patients were reviewed for clinical case discussion with the consultant but were not surgical cases. The waits for the foot and ankle clinics were reduced from 17 weeks to 11 weeks by the introduction of the 3 new weekly clinics.

**Conclusion:** The Stand-alone Foot and ankle triage clinics have been an effective way in reducing the waiting times to be seen by the consultant foot and ankle surgeon. The waits have reduced on average by 6 weeks. The new clinics have provided assessment at an earlier stage and have helped to divert non-surgical cases away from the consultant and get appropriate treatment. Treatment can be started at the first appointment. This shows that the use of appropriately skilled and experienced Podiatrists can help to improve orthopaedic care and can help to create a career pathway for Podiatrists.

### P05 Real time non-instrumented clinical gait analysis as part of a clinical musculoskeletal assessment in the treatment of lower limb symptoms in adults: A systematic review

#### Paul Harradine^1^; Catherine Bowen^2^; Lucy Gates^2^

##### ^1^The Podiatry Centre, Portsmouth, UK; ^2^School of Health Sciences, University of Southampton, Southampton, UK

**Background:** The aim of this review was to evaluate and summarise the current evidence on non-computerised or non-recorded real time adult gait assessment conducted within the clinical musculoskeletal setting. It was hoped a protocol for best practice and a framework for further research could be developed from this search.

**Methods:** Can a protocol for best practice and a framework for further research be established from previous literature relating to non-computerised or non-recorded real time adult gait analysis in a musculoskeletal clinical setting.

A literature review with no limitation on date of publication was conducted on the 18th February 2017.

**Results:** The review found no significantly informative papers relating to the search.

**Conclusion:** The lack of research on the accuracy, reliability and therefore worth of this highly recommended area of musculoskeletal assessment raises concerns over current assessment and treatment pathways. Further work to develop a method by which gait analysis can be routinely employed in musculoskeletal clinics as a diagnostic tool is required, with any new approach undertaking robust methodological testing.

### P06 Effects of foot orthosis adjustment on biomechanical performance with a novel range of pre-formed orthoses designed for the relief of mild intermittent musculoskeletal pain

#### Jennifer Hanning^1^; Stephanie Cooper^1^; Chelsea Starbuck^2^; Victoria Hodgkinson^1^; Carolyn Buckley^1^; Neil Fawkes^1^

##### ^1^Reckitt Benckiser Healthcare, Berkshire, UK; ^2^School of Health and Society, University of Salford, UK

**Background** Stresses on the feet and lower body during prolonged walking or standing can lead to musculoskeletal (MSK) pain and discomfort. A range of six orthotic insoles has been developed to provide unique support, shock absorption, cushioning and pressure re-distribution to various aspects of the plantar surface of the foot in order to treat a variety of lower body musculoskeletal pain related to mechanical stresses. The aim of this clinical trial was to evaluate the effects of foot orthosis adjustment by this range of insoles (full length insole, ¾ length, arch support, heel cup) by measuring their biomechanical impact under the plantar surface in healthy volunteers.

**Methods**: This was a single centre, randomised, open-label, cross-over study evaluating 24 healthy subjects. On a single day, subjects were asked to walk at a self-selected speed during seven conditions (wearing standard shoes alone and six investigational orthotic insoles), which were randomised. In-shoe pressure (F-Scan) and force data were measured to assess shock absorption properties, loading pattern and stability/balance of each insole design versus standard shoes alone.

**Results**: A total of 24 participants were randomised (11 men, 13 women). The mean age was 36.1 years (range: 20 to 55), mean height was 1.695±0.0914 meters, overall mean body weight was 63.63±10.117 kg, and mean BMI was 22.03±2.022 kg/m2. In-shoe dynamic pressure was measured in 23 subjects. Pressure change and pressure time interval measurements were variable between insoles and anatomical areas of interest (ball of foot, medial arch, heel), which was expected given the differing designs of the insoles. Heel peak pressure was reduced across all insoles; a statistically significant reduction was observed in both feet for 4 of the 6 insole designs tested. The most significant changes in pressure time integral were noted in the medial arch area for 5 of the 6 insoles. Furthermore, a decrease in the force time integral was observed in all insoles with statistically significant results observed in either one or both feet. Contact area for the sole and medial arch significantly increased in either one or both feet, for all but one insole tested. No adverse events, adverse device effects or device deficiencies occurred during this clinical investigation.

**Conclusions**: This study has aided in further understanding the biomechanical impact and mode of action of each orthotic design. Insoles with heel cups and medial arch geometries consistently affected peak pressure, pressure time integral and contact area data, conferring a beneficial impact on shock absorption, loading pattern and stability which has the potential to be associated with clinical benefit. A subsequent clinical investigation to evaluate the efficacy of these insoles on specific areas of lower body MSK pain is underway.

### P07 The prevalence and impact of self-reported lower body musculoskeletal pain in a working population

#### Jennifer Hanning; Stephanie Cooper; Adam B Smith; Neil Fawkes; Carolyn Buckley; Victoria Hodgkinson

##### Reckitt Benckiser Healthcare, Berkshire, UK

**Background**: Musculoskeletal (MSK) lower body pain can be caused by long periods of walking or standing and often goes undiagnosed and untreated. The aim of this study was to generate evidence regarding the severity and distribution of self-reported lower body pain in a working population on their feet most of the day and suffering from non-diagnosed pain.

**Methods**: The study was conducted online with a UK sample. Participants with self-reported work-related lower body MSK pain completed the Brief Pain Inventory (BPI), the Work Productivity and Activity Impairment (WPAI) scale, and a quality of life instrument, the AQoL-4D. Information regarding sociodemographics and work patterns was also collected. Statistical analysis was carried out using analysis of variance to assess group differences, and multiple linear regression to explore the impact of BPI on absenteeism/presenteeism.

**Results**: A total of 1035 participants completed the study (57% females). The average age of participants was 43.4 years (18 to 67 years). The majority of participants resided in Scotland (53%) and England (43%). The largest number of participants worked in healthcare (17%) followed by hospital / leisure industry (13%). A large number of participants (60%) had 8+ hour shifts and were on their feet either most or all of the time (78%). Pain was reported most frequently after each shift (27%). Mean pain severity and interference were 4.63 (standard deviation, SD: 2.07) and 4.37 (SD: 2.49) respectively. Mean overall AQoL-4D scores were 0.631 (95% confidence interval, CI: 0.609 to 0.653). The mean number of working hours lost due to MSK pain was 4.12 (SD: 9.93) in those participants who had worked in the previous 7 days; absenteeism and presenteeism due to pain were 10% (SD: 21%) and 33% (SD:25%) respectively. There was strong statistically significant association between the pain measures and absenteeism/presenteeism (p<0.0001).

**Conclusions:** Musculoskeletal lower body pain is highly prevalent in those who spend most of their working day on their feet. This type of pain impacts significantly on their quality of life and productivity at work and has a potential economic impact through lost work and presenteeism.

### P08 A structured literature review on the use of musculoskeletal ultrasound for the evaluation of the Achilles tendon in people with diabetes.

#### Molly Smith^1^; Catherine Bowen^2^; Keith McCormick^2^; Lindsey Cherry^1^

##### ^1^Solent NHS, Southampton, UK; ^2^ School of Health Sciences, University of Southampton, Southampton, UK

**Background**: The Achilles tendon (AT) is an important structure in lower limb function as one of the prime movers during sagittal plane motion. The AT can be pathologically affected by diabetes resulting in reduced function. Musculoskeletal ultrasound (MUS) has been shown to be a reliable, sensitive and specific imaging modality for visualising changes in the AT in healthy volunteers. However, there are few studies detailing the efficacy of using MUS in people with diabetes. The aim of this review was to identify literature related to the use of MUS to image the AT in a) healthy populations and b) those with diabetes.

**Methods**: A PICO (population, intervention/indicator, comparison, outcome) approach was used to define a structured search strategy which included: policy, protocol, proce*, guide*, recommendation*, standard*, technique*, method* pract?e, diabet*, Achilles, “calcaneal tendon”, “tendo calcaneus”, ultraso*. The databases searched were Amed, Cinahl, Cochrane, Pubmed, Scopus and Web of Science from the year 2000 to 2019.

A structured literature review using PRISMA and STROBE guidance was undertaken. After sifting title, abstract and full text sifting, 13 published works met the inclusion criteria for comprehensive appraisal as part of this review.

**Results:** Of the 13 published works, 2 were guidance documents (a guideline and a protocol) and the remaining 11 publications reported the reliability of MUS in people with diabetes (n=4) and without (n=7).The guideline document recommended the foot be placed in prone hanging position with AT measurement in the transverse planes only but does not give justification for this. The protocol document recommended inclusion of evaluation of the contra-lateral limb for comparative purposes and the use of Doppler to measure neovascularisation of the AT. It also recommends AT measurement at 2cm calibration points from the enthesis but does not give justification for this. There was no current literature identified that detailed an optimal MUS protocol to visualise the AT in people with diabetes. Overall, the approach to use of MUS to evaluate the AT in people with and without diabetes varied between studies, lacked reliability testing and reported a range in measurements (0.41-5.2mm).

**Conclusion**: Further work on consensus and reliability is required in this area to establish MUS as an adequate imaging modality for the evaluation of the AT in pathological groups such as diabetes.

### P09 Patient safety: Reducing iatrogenic harm in an acute hospital setting

#### Pauline Johnston; David Wylie

##### NHS Greater Glasgow and Clyde, Glasgow, UK

**Background**: In 2014 The Scottish Foot Action group completed an inpatient audit of patients with diabetes and found that 2.4% had not had their feet checked for risk of ulceration. A follow up campaign called Check, Protect, Refer (CPR for feet) was implemented to raise awareness of the importance of early referral to the MDT/Podiatry team. There was no method available at the time to measure the impact of the campaign on reducing iatrogenic harm. The purpose of this project was to implement a model for improvement which would focus on 3 key areas; Risk assessment on admission, application of pressure redistribution products and immediate referral to podiatry (NICE 2016). A robust method for incidence reporting, supportive peer review and data analysis was designed and implemented from 1st June 2018.

**Method:** The project included 280 wards within NHSGGC. Each ward was provided with information and support of the new system for incidence reporting. The datix system used 8 data entries to determine if the reported incident was avoidable or unavoidable pressure damage. The data was collected and analysed to identify hot spots (areas of 2 or more avoidable incidents within a ward). An action plan and a supportive learning & education plan was put initiated to reduce the potential for future incidents. Further work was done to support the use of pressure redistribution products and mattresses. All wards were provided with information on how to electronically refer to podiatry.

**Results:** From the period of October 2018 – March 2019 a 56.5% reduction trend in incidence in Grade 2 and above iatrogenic harm was recorded. Further data was collected in the repeat 2019 CPR for feet Audit. Significant improvement was found in the 3 key project areas in comparison to the 2014 audit

- Feet checked on admission 96.1% (107% Improvement rate)
- Application of pressure redistribution 70.9% (67.6% Improvement)
- Referrals to Podiatry 76.1% (33% improvement)

**Conclusion:** Although the project is still in its infancy the results after a short period of time are encouraging in demonstrating the impact of applying a robust model of reporting. Developing a positive culture of support has also been of benefit to ward staff by providing a safe and supportive environment for learning and education. This helps to use the learning to prevent future incidents. Investing in the resource to support a prevention model of podiatry care has been shown to be of benefit to the reduction of patient harm and may also bring a cost benefit to the health board.

### P10 Save A Life, Stop A Stroke: Diabetes podiatry and detection of atrial fibrillation (AF)

#### Linda Hicks^1^; Kate Mackay^2^

##### ^1^County Durham and Darlington NHS foundation Trust, Durham, UK; ^2^Academic Health Science Network North East and Cumbria, Newcastle, UK

**Background:** AF is a heart condition that commonly displays no symptoms. Without treatment those living with AF are at increased risk of a stroke costing the NHS on average £23,315 per patient. Patients with diabetes have their pulse checked as part of their annual foot check review to detect the presence or absence of a foot pulse to prevent diabetes complications. The aim of this study is to increase the diagnosis of AF using foot pulse checking during patients diabetes annual foot review.

**Methods:** A three-month pilot (January 2016 – March 2016) was conducted by County Durham and Darlington NHS Foundation Trust and has been part of a wider AF Programme run by the Academic Health Science Network for the North East and North Cumbria (AHSN NENC) in conjunction with the Northern England Clinical Networks. During the initiative, 45 podiatrists were trained to spot heart irregularities, using a Doppler. Any person detected with an irregular pulse was referred to their GP for a 12-lead ECG to confirm diagnosis.

**Results:** 5,000 diabetic patients had their feet pulse-tested. 10 patients with previously unknown AF were detected, indicating that one new case of AF could be identified for every 500 patients. With 1 in 20 patients, either untreated or inappropriately treated, having an AF-related stroke, two patients each year would be prevented from having an AF-related stroke.

**Conclusions:** The pilot has been so successful that the work continues in County Durham and Darlington, and is being spread locally via the Northern Diabetes Footcare Network. If all patients with diabetes, in England, had their pulse checked as part of the annual review screening for an irregular pulse 6800 people could be detected with AF. If all these people were correctly anticoagulated, 340 AF-related strokes could be prevented as well as saving the NHS £7.9M.

### P11 To identify the optimum time needed to detect atrial fibrillation using a doppler

#### Catherine O'Hara

##### New College Durham, NHS CDDFT, Durham, UK

**Background:** Following a review of existing literature, no research had been conducted to determine an appropriate length of time needed to screen a pulse for Atrial Fibrillation (AF) with a doppler. Developments to the understanding of this could contribute to a more effective screening process in podiatry to increase the likelihood of early detection reducing the risk of strokes.

**Methods:** A quantitative methodology based on the positivist paradigm was used to measure a naturally occurring phenomenon using deductive principles. Data was collected from a sample of patients pre-diagnosed with Permanent AF and receiving anti-coagulant therapies.

1. A pilot study followed by 24 separate doppler audio recordings lasting 2-minutes from the dorsalis pedis pulse of 6 participants were collected.
2. An analysis of the 24 pulse recordings measured the length of time it took to confidentially identify an irregular AF pulse.
3. The analysis tool Intraclass Correlation Coefficient (ICC) was used to measure the reliability of both the degree of correlation and the level of agreement from a single-rater to establish the level of intra-rater reliability when the analysis of the recordings was later repeated.

**Results:** Overall, the intra-rater reliability was found to be excellent. There were similar score agreements between ratings producing a strong-positive ICC linear relationship of +0.70. The distribution of results of 65% of the data within the first 10-seconds and 29% of data between 10 and 20-seconds were also noted. These findings show it is possible for a podiatrist to distinguish an AF pulse with a doppler within 20-seconds, 94% of the time. Hypothetically a lengthier time spent on a pulse will ensure quality and consistency in AF detection and consequently reduce risks of AF. All subjects were known to have pre-diagnosed AF by the single-rater, consequently it is recognised that rater-bias could have impacted the data’s validity. This problem often arises when a single-rater acts on behalf of the population. A limitation of this study is human error caused by a delay in the raters reaction time when stopping the stopwatch. This potentially effected the results accuracy, although it is also argued a single-rater will cancel out the overall human error. It is recommended for future research that a larger and a more varied sample is recruited for a blind Randomised Control Trial with a control group of patients with normal sinus rhythm and multiple-raters. This is to eliminate the possibility of rater-bias and to measure inter-rater reliability if variables between raters can be controlled and standardised training for raters received.

**Conclusion:** In conclusion, this primary research contributes to existing knowledge of AF by providing a deeper understanding of the AF screening process in a podiatry setting. This research established that AF detection using a doppler can be achieved in an efficient and practical time within a minimum of 20-seconds and can be feasibly achieved while performing routine podiatry examinations.

### P12 The effect of an diabetic foot ulcer offloading boot (PulseFlow DF) on plantar pressures: a proof of concept study

#### Thomas Dickie^1^; Una Adderley^2^; Anne-Maree Keenan^2^; Graham Chapman^2^

##### ^1^Leeds Teaching Hospitals NHS Trusts, Leeds, UK; ^2^Leeds Biomedical Research Centre, University of Leeds, Leeds, UK

**Background:** Diabetic foot ulcerations (DFU) are consistently considered a chronic wound with increased risk of amputation, infection, suffering and death. It is well established that reducing pressure from the DFU site allows the foot ulcer or damaged tissues to heal. Patient quality of life (QOL) has also been shown to be significantly reduced in diabetic patients with foot ulcerations has compared the physical, emotional and financial impact of DFU as worse than some cancers. The healing of DFU is therefore paramount to avoiding such devastating disease outcomes including amputations, poorer QOL and increased financial burden for the patient and the providing health service. The aim of this study was to provide proof of concept of the effectiveness of a newly designed offloading boot, compared to usual stand of care (USC). Secondary factors related to QOL and patient related factors of using a new device were also explored.

**Methods:** Participants with DFU were recruited from a large diabetic foot treatment centre. Plantar foot pressure was captured using the Pedar-xf analyser in-shoe system during walking: wearing (a) the PulseFlow DF and (b) USC.Pressure time integral (PTI) in the plantar foot were compared. The Novel mask was used to view six recognised regions ofthe foot as practically guided by previous research using the Pedar x analyser insoles^6^.

**Results:** Twelve patients were recruited, (Female 1, Male 11), with a mean age of 65.1years (SD 15.7), diabetes duration of 17 years (SD=13), BMI of 19.5 (SD=6), ulcer duration of 29 weeks (SD=32.1). While there was a difference in the pattern of PTI between the PulseFlow DF and the participant’s USC at the medial forefoot (t=-1.6, p=0.1, 95% CI = - 31.4 to 5.2) and the lateral forefoot (t=-1.3, p=0.2, 95% CI = -24.3 to 6.7), these were not significant.

**Conclusions:** The PulseFlow DF boot alters the PTI in people with diabetic foot ulcers however there is insufficient evidence to suggest it is better than usual standard of care. The use of patient involvement via a questionnaire presented interesting findings i.e. the device was generally acceptable however the lowest median scores related to the device being heavier than expected. This allowed an insight into the use of this new design alongside the aims of the study. However due to the few completed questionnaires (Number=12, fully completed=10) no real statistical analysis can offer strong conclusions. While no difference was found in PTI in the forefoot this could be associated with our small sample size. Further research is required, in the authors' opinion, preferably using a randomised controlled trial using: Primary Aims Time to healing the DFU and Secondary Aims Cost value analysis, Patient involvement in acceptance and time used and Time remained healed.

### P13 Use of negative pressure wound therapy (NPWT) in hard-to-heal diabetic foot ulcers: A challenging patient group

#### Rachael Gilberts^1^; David Russell^1,2^ ; Elizabeth McGinnis^1^; Nikki Dewhirst^1^

##### ^1^Faculty of Medicine and Health, University of Leeds, Leeds, UK; ^2^Leeds Teaching Hospitals NHS Trust, Leeds, UK

**Background:** Negative Pressure Wound Therapy (NPWT) is one of four advanced therapies being investigated in the NIHR HTA funded MIDFUT study, which is looking at the treatment of hard-to-heal diabetic foot ulcers (DFUs). The trial eligibility criteria enables the use of NPWT on a range of wound types which may not always be considered for NPWT in standard practice.

**Methods**: Through practical workshops and discussion of interactive simulated patient case studies in an open forum we have identified and addressed the following considerations for the application of NPWT and potential benefits for this patient group.

**Results**:

1. Wound area - NPWT can be used for wounds from 0.8cm2 and above, wounds of this size are not ‘too small’ to be considered for NPWT application
2. NPWT can be used in high sheer/friction locations on the foot with use of black foam which is designed for high pressure areas. Appropriate offloading devices can be used with NPWT in place
3. Many areas of the foot are suitable for NPWT to be applied. Bridging and other techniques are available for wounds in ‘difficult’ locations and full training on these specialist techniques are provided
4. NPWT is effective for management of heavily exudating wounds, however there are other benefits including encouraging angiogenesis and NPWT can also be applied to wounds which appear ‘dry’
5. NPWT is used most prominently on acute wounds. There is a lack of robust evidence for the use of NPWT on chronic DFUs and the MIDFUT trial will help to answer this question
6. Changes in NPWT technology have been made to enhance patient experience and acceptability
7. Patient equipoise can be challenging, particularly if previous experience has been unfavourable. Consideration to patient approach and the Quintet technique [1] is recommended
8. Liaison with community teams for dressing changes between clinic visits is important. Providing a letter to patients which can be shown to all Registered Healthcare Professionals involved in their care package is beneficial for a multi-disciplinary approach to patient care

**Conclusion:** Through close liaison with clinicians and successful training workshops we have developed guidance for the use of NPWT in a chronic hard-to-heal DFU population which may not previously have been considered to receive this intervention. This guidance is discussed with participating centres in detail at the site initiation visit and intervention training sessions to enable development of NPWT application skills and ensure all eligible patients have the opportunity to take part in research.

### P14 Clinical academic careers -- what are they, are they for me & how do I get on this career path?

#### Lindsey Cherry^1^; Anne-Maree Keenan^2^

##### ^1^School of Health Sciences, University of Southampton, Southampton, UK and Solent NHS Trust; ^*2*^*Leeds Biomedical Research Centre, University of Leeds, Leeds, UK*

**Background**: The National Institute for Health Research in partnership with Health Education England provide funded opportunities for Podiatrists to learn about research whilst continuing to develop their clinical practice.

**Methods:** There is a range of educational funding streams available for podiatrists of all backgrounds, ranging from internships for those who are perhaps newly qualified through to post-doctoral awards for those more experienced in research. Podiatrists at all clinical levels with an ambition to make improvements to the service they provide are able to learn about how to challenge convention or discover new solutions through these awards. However, identifying the right opportunity for the individual or navigating the application process can be daunting.

**Results:** For clinical managers there may be competing resource demands and releasing staff to undertake personal awards can be a challenge. There is a need to identify key networks to support applications, including academic mentors (typically in a university setting) and clinical leads. These challenges are current but not new and there are some examples of how they have been overcome. The NIHR Podiatry Advocates are experienced in providing supporting information to help address some of these difficulties (https://www.nihr.ac.uk/our-research-community/NIHR-academy/career-development-support/nihr-training-advocates.htm). The Advocates provide signposting to either individuals, clinical managers, academics or teams in order for them to submit high quality applications. Those who see challenges within their clinical work but are keen to make an improvement are encouraged to consider whether there is an NIHR funding stream available to support them.

**Conclusion:** HEE/NIHR offer a range of training opportunities for Podiatrists to get involved in research and improvement. The Advocates are experienced in supporting people or teams to identify and apply for funding.

### P15 Raising awareness of heel pressure damage within York and Scarborough hospital

#### Claire Davies^1^; Samantha Haigh^2^; Lisa Pinkney^2^

##### ^1^Harrogate and District NHS Trust, Harrogate, UK; ^2^York Teaching Hospital NHS Trust,York, UK

**Background:** Pressure ulcers are a key indicator of the quality and experience of patient care. 700,000 people are affected by pressure ulcers each year. Treating pressure ulcers costs the NHS more than £3.8 million every day. Many pressure ulcers are preventable, and when they do occur they can have a profound impact on the overall wellbeing of patients, and can be both painful and debilitating. Heel ulcers are one of the most challenging areas of pressure ulcer management, as not only are they the second most common site for pressure ulceration, they can sit alongside other complicated conditions which affect the feet, such as diabetes and peripheral vascular disease. This can make them both confusing and difficult to diagnose and treat.

The Trust was invited to participate in an improvement project set up as part of the national NHS Improvement’s Stop the Pressure collaborative. The area of focus in the Trust was on heel pressure ulcers, as baseline figures from Trust data showed this to be the most problematic and difficult to manage area. The project aim was to reduce hospital acquired heel pressure ulcers by fifty per cent over six months.

**Methods**: The PDSA took place across two hospital sites, focusing on four Elderly Medical Wards and included increasing staff education, suportive visual aids, increased provision of offloading devices on wards, and promotion of shared care between patients and staff. This was a collaborative approach with representatives from podiatry, tissue viability and patient safety teams.

The visual aids were developed by the inpatient podiatrist and formed a key part of the education for staff and patients. The choice of subjects for visual aids came from common themes from root cause analysis investigations and recurring findings on the wards: Ward staff not confident in categorising of heel pressure ulcers defaulting to unstageable. Soft casts being applied incorrectly or thrown away, increasing risk of cast rubs and significantly duplicating podiatry workload. Ward staff attempting to use offloading methods such as pillows to float heels but often using them incorrectly or only using them when damage had occurred. Patient non-concordance, often linked with not understanding the reasons behind an action or treatment.

**Results:** The project findings were not what the team expected. Numbers did not reduce significantly over six months and only small changes were identified in reduction of heel ulcers, partly because numbers were relatively small to begin with. However, the team felt that the impact which came about from other aspects of the project were just as important.

**Conclusion:** Increased staff knowledge and awareness around heel pressure damage, patient empowerment to help reduce their own risks of pressure ulcers, significantly quicker time from identification of problem to treatment for patients through earlier detection and the set up of electronic referrals to podiatry, improved accuracy of pressure ulcer categorisation, 100% reduction in misuse of softcasts by ward staff and more joined up collaborative working between the podiatry, tissue viability and patient safety teams.

### P16 Normative values for protective sensation of foot using Neurotouch device in healthy individuals

#### G. Arun Maiya

##### Centre for Diabetic Foot Care and Research, MAHE University Manipal, India

**Background:** Type 2 diabetes mellitus (T2DM) is one of the fastest growing non-communicable diseases worldwide and its prevalence is increasing rapidly around the world parallel to increase in the obesity. Foot ulcers, infections, and deformity are some of the major sources of mortality and morbidity among the population with type 2 diabetes mellitus. Assessment of protective sensation using Smith Weinstein 10g monofilament is part of routine clinical practice. However, there is no objective method of measuring protective sensation in people with diabetic peripheral neuropathy. Therefore, the objective of the study is to determine the normative values for protective sensation of foot using Neurotouch device in Indian Population.

**Methods:** After obtaining institutional ethics committee clearance and informed consent, the detailed foot assessment was conducted on 390 healthy participants using NEURO TOUCH device, which incorporates Monofilament, Vibration perception threshold, Hot and cold perception and Infrared thermometry in a single handheld battery-operated instrument. The protective sensation was measured using monofilament module of Neurotouch which shows objective reading of the protective sensation measured in grams(gms).The data was statistically analyzed.

**Results:** The results showed that out of 390 healthy participants, protective sensation values were less than 10grams (N= 60) between 10-20grams (N=316), and more than 20grams (N=14). The mean values were shown as **11.9±4.5** for the protective sensation. However with existing 10gm. However, in the exiting Protective sensation (10 g Semmes-Weinstein monofilament), we report protective sensation as either present or absent. However, with new Neurotouch device, along with patient reported finding, we will be able to record the protective sensation in grams.

**Conclusion:** In the present study, we established the normative values for protective sensation of foot in healthy individuals using Neurotouch device. The present study concluded that the normative values for protective sensation is found to be in the range of 10-20grams and provides a substantial evidence to stratify the people with diabetic neuropathy based on monofilament readings using Neurotouch device.

### P17 Transforming home visit foot health service to eliminate waits and improve patient safety

#### Laura Price

##### Guy's and St Thomas' NHS Foundation Trust, London, UK

**Background:** The home visit service had acrued long waits for housebound patients to be seen by the Foot Health Team. This generated complaints by patients and highlighted a risk in delay for the most high risk patients who were vulnerable to foot health deterioration. The aims of the two year project were to:

- See all home visit patients for review when we said they'd be seen
- To prioritise urgent high risk to be seen within 48 hours
- Ensure all new patients were seen on time
- Reduce overall caseload by redirecting non eligible patients to the most appropriate clinic, low risk patients to be sign posted to social care or self care where appropriate
- Better liason with acute MDT for earlier referral and intervention for high risk patients who are at risk of limb loss/ further deterioration.

**Methods:** Actions taken during the project were:

- Reassessment of the whole caseload of housbound patients to ensure eligibility criteria was followed. Improving the eligibility criteria and development of mobility criteria to identify patients that can transfer to clinic for ongoing care.
- Utilising feedback from home visit Podiatrists, using suggestions boxes and annoymous feedback to shape the project. Staff involved at every step. Better liason with District nurses, neighbourhood nurses and GPs informing them of changes to foot health and collaboration at an earlier stage to ensure early intervention for patients.
- Employment of a podiatry assistant to aid seeing moderate and high risk nail care patients in a timely manner to ease pressure off main caseload.
- Prioritising home visit sessions to maintain capacity especially during sickness and annual leave.

**Results:** The project lead to a dramatic reduction in inappropriate referrals and streamlined demand. As a result, capacity has been closely matched to true demand allowing us to respond quickly to high risk patients requiring a visit within 48 hours. Pre transformation in 2016, 1349 patients out of a caseload of 2217 were overdue. The longest wait time being 65 weeks.

Post transformation in 2019 zero patients were waiting longer than their follow up review date and all urgent referrals are seen within 48 hours. All new referrals are seen on time and there has been zero complaints in the last 8 months.

**Conclusion:** Plans for the sustainability of this work include, audit of service standards for high risk patients to ensure they are seen within 48 hours of referral and maintain zero waits for all follow up patients. To audit the effectiveness of seeing all patients on time against re-ulceration rates for high risk wound patients. Increase clinical supervision of Band 5 and 6 home visit podiatrists to increase wound care knowledge and to ensure quality and safety of all patients.

### P18 Quality improvement project a pilot study of a high risk podiatry service model introduced into a care home

#### Natalie Roe

##### City Hospitals Sunderland, NSH Foundation, Sunderland, UK

**Background:** This report aims to demonstrate how a podiatry service quality improvement project into a residential care home was implemented and why it was needed. Risk of foot ulceration increases in those with age, neuropathy, peripheral vascular disease and immobility. In 2013 the Sunderland Care Commissioning Group piloted, through the readmission scheme, a podiatry service providing specialist care into 11 care homes in the Coalfield’s locality of Sunderland, which are a mixture of both nursing and residential homes. The service provides, foot care for all residents and prevention strategies for those classed at risk of developing foot ulceration and to provide specialist wound care interventions for residents presenting with foot ulcers into each care home in the Coalfield’s locality. There are financial constraints to rolling out the Coalfield’s model to the further 36 care homes in Sunderland. Therefore, a new model was explored. This is a high-risk model only, piloted in one care home.

**Methodology:** Driver diagram was used to define key problems, activities required to deliver improvement. Fish bone diagram was used to establish the route cause analysis. The plan do study act (PDSA) cycles were used to pilot the interventions at the study site to ensure effective small change The Comb-B Behaviour Change Wheel was also included to ensure that interventions undertaken were sustainable by identifying the behavioural change required.

**Results:** There were no pressure injuries identified at post-intervention, this was a significant improvement from baseline. Staff had referred three grade 1 pressure injuries and one foreign body, one foot pain and two trauma ulcers during the intervention, showing a 300% increase in reported grade 1 pressure injuries.

**Conclusion:** This quality improvement project has demonstrated a person centred, evidence based, older person service delivery for sustainable future that embeds a multi-disciplinary team to the high-risk podiatry model of service. It has shown that behavioural change has occurred with small change interventions. It has used the robust methodology of PDSA to effect this change and allow for a clear report to be able to articulate the benefit.

### P19 Skin surface pH of the healthy adult foot

#### Jennifer Andrews; Carina Price; Farina Hashmi

##### School of Health and Society, University of Salford, Salford, UK

**Background**: The stratum corneum is the uppermost layer of the epidermis and is covered by a thin layer of sweat, sebum and epidermal lipids that are collectively called the 'acid mantle'. The low pH (between 4 and 7) of this surface contributes to the integrity of the stratum corneum, reduces permeability and reduces proliferation of potentially hazardous micro-organisms on the skin surface. The pH of skin on the foot is poorly understood but the limited information we do have suggests that the foot skin surface is significantly different to the rest of the body.

**Method**: 28 healthy participants with no history of foot skin pathology were recruited to have the skin pH measured at nine different locations on the foot using a glass electrode pH probe (Dermalab Combo, Cortex, Hadsund, Denmark).

**Results**: Statistically significant differences were found between the skin surface pH at different sites on the foot. The pH measurements were significantly less acidic on the plantar skin sites (median: 5.61) compared to the dorsal sites (5.02, P<0.001). The plantar surface medial longitudinal arch, however, displayed a pH measurement more comparable to the dorsal surface measurements.

**Conclusion**: Further investigation is required to explore the significance of these data in regards to the immune and barrier function of the skin at these different locations.

### P20 Back to basics

#### Cheryl Baillie^1^; Leanne McGennity^1^; Georgia Carlington^1^; Jill Cundell^2^

##### ^1^Belfast Health and Social Care Trust, Belfast, Northern Ireland; ^2^Ulster University/ Belfast HSCT, Belfast, Northern Ireland

**Background:** ‘Health and Wellbeing 2026; delivering together’ advocates an approach to achieving better outcomes for individuals and populations. It is well documented within this framework that to achieve this we need to continuously improve on the quality and safety of care. Clinically for podiatry, this means ensuring patients are assessed in a timely and appropriate way in line with regional and national guidelines and thus this ‘basic’ information directly impacts on the provision of care for our service users and the delivery of our service. Ultimately the basics are important to ensure safe practice, standardised care, and to formulate treatment plans tailored to the individual’s needs, to ensure assessment of our patients is meeting current regional and national guidelines and professional accountability.

**Methods:** To determine if a patient meets the podiatry service access criteria an assessment of the individual’s risk status is required. This also helps us identify the patients RISK of developing potentially limb-threatening foot complications. This information is also vital for audit, future workforce planning and capacity management our service. To carry out this project we used the model for improvement framework. Our Health and Social Care Trust's community podiatry service is based in 6 clinical locations, all providing a service in the clinical and domiciliary setting. A random sample was identified from each setting using random.org. The information for 60 patients was selected, 10 from each location.

**Results:** From our sample, 100% of assessments and risk assessments were open of our 60 patients but only 60% had been risk assessed within a 12 month period. So whilst everyone had been risk assessed our guidelines state that this should be completed again within a 12 month period so we are falling below our target of 100%. We found 73% of vascular assessments from our sample of 60 have been completed within the recommended 12 month period and 70% of the neurological assessments were completed within the 12 month period. Staff (n=70) were surveyed using a questionnaire, to see if we could identify reasons why the completion of assessments and assigning of risk. 15 of those surveyed responded all stated they understood the importance of completing assessments and assigning risk. Barriers identified to doing this included time constraints, unaware that assessments had to be completed within 12 months and some of the tabs on the electronic record system were deemed not to be user-friendly.

**Conclusion:** Changes that have been/are being put in place include:

- Currently, the advanced Podiatrists are working on Competency documents which will be used to carry out a more structured supervision process for all clinicians.
- Wound HUDDLEs are in place in both hospital and community clinics –supporting clinicians in their clinical decision making and helping develop the correct treatment pathways for patients.
- Those surveyed suggested a prompt to remind staff to update assessments and assign risk. As a result, we have devised ‘Think Risk’.

In the future we will evaluate the changes put in place to determine if they have had an impact on the provision of care we provide.

### P21 Under Pressure

#### Michele Doherty^1^; Linda Paine^1^; Patricia Smyth^1^; David McKeown^1^; Jill Cundell^2^

##### ^1^Belfast Health and Social Care Trust, Belfast, Northern Ireland; ^2^Ulster University/ Belfast HSCT, Belfast, Northern Ireland

**Background:** This service quality improvement project aimed to develop an offloading pathway to ensure timely, consistent and appropriate offloading therapy for foot ulceration in our Health and Social Care Trust (HSCT). It is well established that neuropathy, deformity and trauma are the most common causes of Diabetic Foot Ulceration (DFU). Evidence is clear that adequate off-loading increases the likelihood of DFU healing and that increased clinician use of effective off-loading is necessary. However recent surveys show a large discrepancy between guidelines and clinical practice in off-loading diabetic foot ulcers.

**Methods:** An audit was conducted in 2015 of offloading carried out within the high-risk patient group in BHSCT, and this had indicated that offloading was not being optimised - 43% of patients had been offloaded v 57% who were not offloaded. This clearly highlighted variations in clinical practice. Using the PDSA cycle as our model a questionnaire was devised to determine if staff had the relevant baseline knowledge expected prior to implementing a new pathway. Following analysis of the audit findings training on offloading was provided for all staff in both hospital and community services and a further questionnaire administered post this.

**Results:** The results showed that the training provided had increased staff knowledge and confidence in providing various offloading devices for high-risk patients. We recognise that training needs to be ongoing to ensure offloading is embedded in clinical practice. We then developed an offloading pathway document, using the Welsh National Plantar Ulceration Off Loading Pathway 7as a model. This first draft is now ready for consultation. Following consultation, there will be a rollout and implementation of the new pathway, followed in time by an audit to assess adherence.

**Conclusion:** After the implementation of the new pathway further planned steps include a study to investigate the effects of offloading on ulcer recurrence rates within our Health and Social Care Trust and the introduction of the pathway at a regional level. Within BHSCT future steps for this quality improvement project will also include:

- Standardisation of offloading devices available through our Health and Social Care Trust
- The development of an eLearning programme which will be mandatory for all podiatry staff
- Regular re-auditing to ensure consistent offloading knowledge and use
- Include offloading training for all new staff as part of the induction programme

### P22 Assessment and management of symptomatic, idiopathic pes planus in children: a synthesis of UK-based allied health professional practices

#### Stewart C. Morrison^1^; Madeleine Tait^1^; Kyra Kane^2^; Chris Nester^3^

##### ^1^School of Health Sciences, University of Brighton, Brighton, UK; ^2^Wascana Rehabilitation Centre, Saskatchewan Health Authority, Wascana, Canada; ^3^School of Health and Society, University of Salford, Salford, UK

**Background:** Children with symptomatic idiopathic pes planus frequently present to health professionals with foot pain, functional impairment, and proximal joint problems. Evidence underpinning treatment effectiveness for this condition remains elusive and work to explore current clinical approaches is needed. **Aim:** This study sought to explore the perspectives of three professional disciplines on the assessment and management of symptomatic, idiopathic pes planus in children.

**Methods:** Data was collected from UK-based podiatrists, physiotherapists, and orthotists with experience of managing foot problems in children. Data was collected via a self-administered online survey and data was captured over a four-month period from July – October 2018. The project was approved by the institutional ethics panel and all participants provided informed consent.

**Results:** Fifty-five participants completed the survey: 33 physiotherapists, 16 podiatrists, and 6 orthotists. The results revealed that assessment techniques varied between professions with standing tip-toe (podiatrists - 62.5%; physiotherapists - 51.5%; orthotists – 66.7%) and joint range of motion (podiatrists - 68.8%; physiotherapists - 36.4%; orthotists - 83.3%) being the most commonly used. Professionals reported using a diverse range of interventions. Forty percent of all respondents used orthoses (pre-fabricated or custom) as a first-line intervention; podiatrists tended to use prefabricated orthoses (81%). There and there were no statistically significant differences between professions (p = 0.07). Sixty-three percent of respondents reported that resolution of symptoms was the key marker for withdrawal of intervention but acknowledged that the decision-making process was complex. Use of child-specific, standardised measures to evaluate outcomes in practice was variable.

**Conclusion:** These results illustrate inconsistencies between allied health professionals with respect to assessment, management, and outcome measurement for symptomatic paediatric pes planus. These findings might be explained by the lack of robust evidence to guide practice and suggest a need for further work to harmonise assessment and treatment approaches between professions. Addressing these discrepancies could help prioritise professional roles in this area, and better support the management of these children through evidence-based, cost-effective interventions.

### P23 Orthotics for treatment of symptomatic flat feet in children a randomised controlled trial - the OSTRICH study

#### David Torgerson^1^; Sarah Cockayne^2^; Joy Adamson^2^; Mike Backhouse^2^; Caroline Fairhurst^2^; Catherine Hewitt^2^; Anne-Marie Keenan^3^; Stewart Morrison^4^; Daniel Parker^5^; Daniel Perry^6^; Sarah Ronaldson^2^; Tim Theologis^6^

##### ^1^York Trials Unit, University of York, York; ^2^Health Sciences and York Trials Unit, University of York, York; ^3^ Leeds Biomedical Research Centre, University of Leeds, Leeds, UK; ^4^University of Brighton, Brighton, UK; ^2,5^School of Health Sciences, University of Salford, Salford, UK; ^6^University of Oxford, Oxford, UK

**Background:** Pes planus have been described as one of the most common conditions seen in paediatric practice (1,2) and the most common reason for attending paediatric orthopaedic clinics (3) but questions remain about the most effective treatment modality, or whether treatment is even necessary. The aim of this study is to evaluate the clinical and cost-effectiveness of custom-made and prefabricated orthoses in addition to exercise and advice, compared with exercise and advice alone on the physical functioning of children with symptomatic pes planus.

**Methods:** OSTRICH is a multi-centre, three-armed, pragmatic, individually randomised, controlled trial. It includes an internal pilot, economic and process evaluation and qualitative study. We plan to open 30 recruiting sites from any primary or secondary care NHS outpatient clinic providing care for children with pes planus. We will recruit 1085 children/young people aged between six and 16 years, who have symptomatic pes planus. Participants will be excluded from the trial if there is a history of major trauma to the lower limb; pes planus of non-idiopathic cause or symptoms secondary to any systematic condition/syndrome/malignancy; have a history of foot and/or ankle surgery or require an ankle-foot orthoses or other lower limb device.

Participants will be randomised in a 2:2:1 to custom orthoses, prefabricated orthoses both with exercise and advice or exercise and advice alone. The type of orthoses used in the study will be informed by a clinician survey and consensus meeting of clinicians taking part in the trial. The primary outcome is the physical domain subscale of the Oxford Ankle Foot Questionnaire (OxAFQ). Secondary outcomes include: school and Play, Emotional and Footwear subscales of the OxAFQ; EQ5D-Y, text message pain scores. Costs and outcomes will be measured at baseline, three, six and 12 months. These will be collected using a combination of postal questionnaires and medical records.

**Results:** The internal pilot study will include qualitative interviews with children and parents and clinicians to highlight any barriers or facilitators to recruitment of trial participants. Treatment fidelity will be assessed using the following methods: 30 observations of participating clinicians; case report forms; patient reported adherence and interviews with trial participants to determine the acceptability of the intervention. Our implementation strategy will be informed through two focus groups with participating clinicians which will discuss possible barriers and facilitators to implementing our findings in routine practice.

**Conclusion:** *Timescales:* Recruitment to the pilot trial is due to start in December 2019 at five pilot sites. We will progress to the main trial in June 2020 with follow up of participants due to end in June 2022. The results of the study will be available in November 2022.

